# Advanced solar radiation prediction using combined satellite imagery and tabular data processing

**DOI:** 10.1038/s41598-025-96109-0

**Published:** 2025-04-23

**Authors:** Mohammed Attya, O. M. Abo-Seida, H. M. Abdulkader, Amgad M. Mohammed

**Affiliations:** 1 Department of Information System, Faculty of Computers and Information, Kafrelsheikh University, Kafrelsheikh, Egypt; 2 Department of Computer Science, Faculty of Computers and Information, Kafrelsheikh University, Kafrelsheikh, Egypt; 3 Department of Information System, Faculty of Computers and Information, Menoufia University, Menoufia, Egypt

**Keywords:** Solar radiation, LSTM, GANs, Identity block, Latent diffusion model, Computer science, Environmental impact, Energy management, Energy efficiency, Sustainability, Energy and behaviour, Applied mathematics, Information technology

## Abstract

Accurate solar radiation prediction is crucial for optimizing solar energy systems. There are two types of data that can be used to predict solar radiation, such as satellite images and tabular satellite data. This research focuses on enhancing solar radiation prediction by integrating data from two distinct sources: satellite imagery and ground-based measurements. By combining these datasets, the study improves the accuracy of solar radiation forecasts, which is crucial for renewable energy applications. This research presents a hybrid methodology to predict the solar radiation from both satellite images and satellite data. The methodology basis on two datasets; the first data set contains tabular data, and the second dataset contains satellite images. The framework divides into two paths; the first path take the input as the satellite images; this stages contains three steps; the first step is removing noise using latent diffusion model, the second step is about pixel imputation using a modified RF + Identity GAN (this model contains two modification the first modification is adding the identity block to solve mode collapse problem in the GANs and the second modification is to add the 8-connected pixel to generate a value of missing pixel near to the real missed pixel. The third step in the first path is about using the self-organizing map to identify the special informative in the satellite image. The second path take the input as tabular data and use the diffusion model to impute the missing data in the tabulated data. Finally, we merge the two path and use feature selection to be as input for the LSTM for solar radiation predictions. The experiments done prove the efficiency of the used stage such as missing pixel imputation, removing noise, missing data imputation and prediction using LSTM when compared with other available techniques. The experiments also prove the enhancement of all prediction model after adding two paths before the prediction step.

## Introduction

Solar radiation is a free and invaluable resource for numerous sectors, including heat, health, tourism, agriculture, and energy production^1^. It plays a vital role in the sustainability of biological and chemical processes in nature. Regional satellite images and satellite-derived tabulated data have become essential tools in predicting solar energy radiation. Satellite images provide detailed visual information on cloud cover, atmospheric conditions, and surface features, which are crucial for accurately forecasting solar radiation^2^. Additionally, satellite-derived tabulated data offers precise measurements of solar irradiance and related atmospheric variables^3^. By integrating these two sources of information, we can enhance the accuracy of solar radiation predictions, ultimately improving the management and utilization of solar energy resources across various sectors^4^.

Accurate prediction of solar radiation through satellite data become a trendy area in the field of renewable energy and computer science. Recently many algorithms and models have designed and used in this area. Techniques for predicting solar radiation have evolved significantly, leveraging advanced technologies and methodologies^5^. Traditional approaches often relied on ground-based measurements and empirical models, which, while useful, had limitations in spatial coverage and accuracy^6^. Modern techniques now incorporate remote sensing technologies, such as satellite imagery and satellite-derived data, to provide comprehensive and precise information on atmospheric conditions, cloud cover, and surface reflectance^7^. These methods utilize sophisticated algorithms and machine learning models to analyze the data, leading to improved accuracy in solar radiation forecasts. By combining satellite data with meteorological information and historical solar radiation patterns, these predictive models can offer reliable and detailed insights, enabling better planning and management of solar energy resources^8^.

Despite their valuable contributions to predicting solar radiation, regional satellite images and satellite-derived tabulated data have inherent limitations^9^. One significant issue is the presence of noise in satellite images, which can arise from various sources such as sensor errors, atmospheric disturbances, and signal processing artifacts. This noise can obscure important details and reduce the accuracy of solar radiation predictions^10^. Additionally, satellite images often suffer from missing pixels due to factors like cloud cover, shadows, or technical glitches in the imaging sensors. These gaps can create challenges in obtaining a complete and continuous dataset^11^. Similarly, satellite-derived tabulated data can experience missing data points due to transmission errors, satellite malfunctions, or temporal gaps in data collection. These missing values can hinder the reliability of the predictive models and require sophisticated data interpolation and correction techniques to mitigate their impact^12^. Another critical limitation is the challenge of feature extraction from satellite images, where identifying and isolating relevant features such as cloud patterns and surface reflectance can be complex and computationally intensive^13^. Addressing these limitations is crucial for enhancing the precision and dependability of solar radiation prediction models.

Satellite images are prone to various types of noise and missing pixels that can compromise their quality and utility^14^. Sensor noise, caused by the inherent imperfections in the imaging sensors, results in random variations in pixel values^15^. Atmospheric noise, due to the scattering and, absorption of signals by atmospheric particles and gases, leads to distortions. Quantization noise occurs during the digital conversion of analog signals, causing a loss of fine details^16^. Compression artifacts, introduced when images are compressed, can produce blockings or blurring^17^. Additionally, striping noise, characterized by parallel lines across the image, stems from calibration errors or sensor inconsistencies. Missing pixels, resulting from cloud cover, shadows, or technical issues, create gaps in the dataset^18^.

This research leverages Long Short-Term Memory (LSTM) networks for accurate solar radiation prediction after solving the problems in the preprocessing step for both tabulated data and imaginary data such as removing noise, missing data imputation and features extractions.

The main contribution of this paper as the follow:

- The research presents a framework basis on both satellite tabular data and satellite image overcomes the other research which basis on one of them, the paper basis in different types of features compared to other the previous papers in this area.
- The research introduces two modifications in RF + GANs; the first modification is to use the identity block to solve the problem of mode collapse in the traditional GAN; the second modification is to add the 8-connected pixel, that help to produce a more accurate generated pixel which allow the integrity between the generated pixel and the other pixel of the images.
- The modification of RF + GANs helps to solve the mode collapse in the traditional GANs.
- The paper achieves good results in removing noise, missing pixel imputation and prediction solar radiation compared to the other methods and the preprocessing layer helps to enhance the accuracy of the different types of models for prediction of solar radiation.

The remaining parts of the paper organized as the follow:

“Related Work” presents the related work, “Proposed methodology” presents the proposed methodology, “Results” presents the results and “Conclusion and future work” presents the conclusion and future work.

## Related work

Accurate prediction of solar radiation is a critical challenge in optimizing solar energy utilization. Traditional approaches have relied on empirical models and ground-based measurements, which, although useful, suffer from spatial limitations and sensitivity to environmental fluctuations^19^. To overcome these challenges, researchers have explored advanced computational techniques, including artificial intelligence and machine learning, to improve the accuracy of solar radiation forecasting^20^.

One of the earliest methods involved using statistical regression models combined with meteorological data to estimate solar irradiance. Wang et al.^21^ proposed a series of global radiation models that integrated atmospheric parameters to enhance prediction accuracy. Similarly, Gopal et al.^22^ employed satellite-derived reflectance data to estimate solar exposure, demonstrating how remote sensing technologies can supplement traditional ground-based measurements. However, such methods often struggle with incomplete data and require sophisticated imputation techniques to maintain prediction reliability^23^.

Machine learning and deep learning models have been increasingly applied to solar radiation forecasting due to their ability to learn complex nonlinear relationships from diverse data sources. Zhu et al.^24^ developed a recurrent neural network model that combines meteorological variables with historical solar radiation data, achieving significant improvements in long-term predictions. Acikgoz et al.^25^ demonstrated the efficiency of hybrid deep learning approaches by fusing convolutional and recurrent networks for enhanced feature extraction in solar energy applications. These models, while powerful, require large datasets and careful preprocessing to avoid overfitting and maintain generalization across different climatic regions^26^.

Remote sensing techniques have also played a crucial role in improving solar radiation forecasting by providing high-resolution data on atmospheric conditions. Kotthaus et al.^27^ reviewed various remote sensing methodologies for estimating ground-level solar irradiance, highlighting the impact of cloud movement and aerosol concentration on radiation levels. Coruhlu^28^ introduced an environmental monitoring approach that integrates satellite imagery with land-use classification models to refine solar energy potential assessments. These methods underline the necessity of integrating spatial and temporal factors for accurate solar forecasting^29^.

A major limitation in solar radiation modeling is the presence of noise in satellite images, which can distort predictions. Yuzer et al.^30^ explored the inherent uncertainties in satellite-based remote sensing, emphasizing the importance of filtering techniques to reduce errors in solar radiation estimation. Similarly, Ajith et al.^31^ investigated various image processing techniques to enhance cloud classification, demonstrating how improved image clarity leads to more accurate irradiance predictions. Such advancements in data preprocessing contribute to more robust and reliable solar radiation forecasting models^32^.

Recent studies have also explored the potential of generative models to address missing data issues in solar radiation estimation. Ghildiyal et al.^33^ utilized adversarial learning techniques to synthesize realistic satellite data, improving the robustness of predictive models. Koochali et al.^34^ extended this work by integrating probabilistic frameworks that account for data uncertainties, leading to more stable long-term forecasts. These approaches highlight the growing role of generative models in handling data sparsity and enhancing predictive accuracy in solar energy applications^35^.

Building on these advancements, this research proposes a hybrid framework that integrates machine learning, deep learning, and generative models to improve solar radiation forecasting. The methodology incorporates advanced feature extraction, noise reduction, and missing data imputation techniques to enhance prediction accuracy. By leveraging both satellite-derived imagery and meteorological datasets, this study aims to develop a more comprehensive and reliable model for solar radiation prediction. Table 1 provides an overview of recent studies in the field, outlining their methodologies, accuracy, and limitations.

**Table 1:** A list of related studies that used in prediction of solar radiation with their limitations.

Article	Year	Techniques Used	Description	Accuracy	Limitation
Nespoli et al.^36^	2022	Hybrid approach	Integrated satellite imagery and tabular data for solar radiation prediction	Moderate	Sensitive to noise and missing data
Huang et al.^37^	2023	LSTM + CNN	Fusion of infrared cloud images and radiation data for solar prediction	High	High computational cost
Cheng et al.^38^	2021	CNN	Short-term solar power prediction using regions of interest in satellite images	High	Requires large-scale training data
Ahn et al.^39^	2024	HRNet model + satellite data	Enhanced short-term solar radiation prediction	High	Computational complexity
Hayawi et al.^40^	2024	Data imputation + ML	Climate data imputation and improvement for solar radiation estimation	Moderate	Sensitive to outliers
Li et al.^41^	2023	WGAN + LSTM	Hybrid generative and sequence learning model for irradiance forecasting	High	Lacks real-time adaptability
Zhao et al.^42^	2022	ML regression models	Comparison of multiple ML models for satellite-based prediction	Moderate	Limited generalization to new regions
Irshad et al.^43^	2023	Hybrid DL model	Deep learning-based hybrid method for hourly solar radiation forecasting	High	Computationally intensive
Han et al.^44^	2023	Latent diffusion model	Noise reduction in satellite images for improved prediction	High	Loss of fine image details

## Proposed methodology

This part of the paper presents the methodology and each part of it. The methodology is based on two types of data: satellite images and satellite-derived tabular data. The problem of solar radiation prediction can be formally defined as follows: Let (x) be the input data, which consists of satellite images and tabular data (e.g., meteorological parameters). The goal is to predict the solar radiation (y) at a given time and location. Our proposed method (f) takes (x) as input and produces (y) as output, i.e., (y = f(x)). Here, (f) represents our hybrid model, which includes preprocessing steps (e.g., noise removal, missing data imputation) and a modified LSTM for prediction. Our approach is a forecasting model, as it uses historical data (e.g., past satellite images and meteorological data) to predict future solar radiation values.

**Fig. 1:**
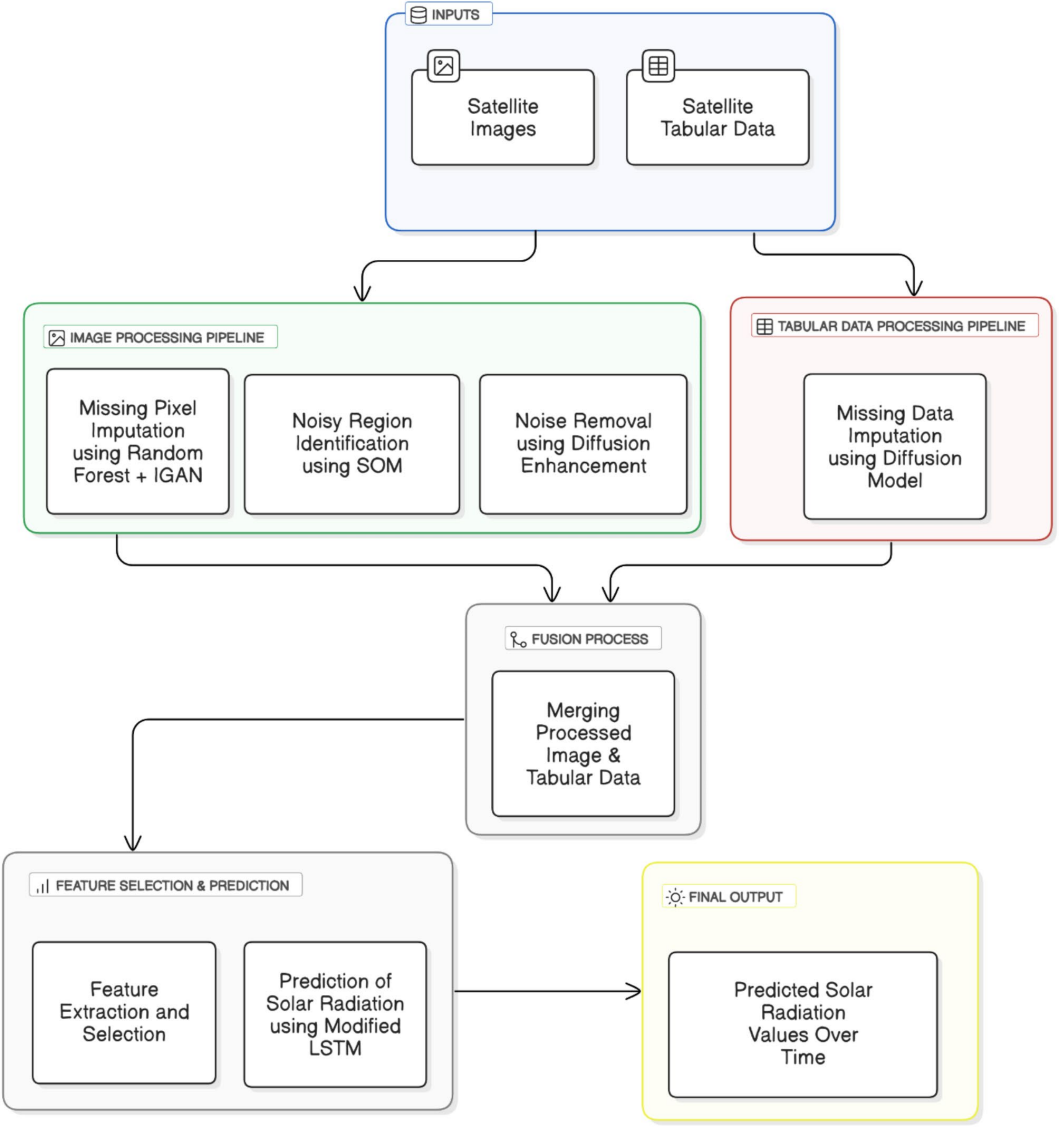
The methodology diagram of solar radiation prediction.

The methodology contains two paths: the first path takes the input as the satellite images, and the second path takes the input as satellite-derived tabular data. The first path contains three main steps: the first step is about missing pixel imputation by imputing the missing pixel using random forest and identity GANs. The paper presents three novel modifications in this step: the first modification is about adding the identity block to the generator in the GANs to avoid the vanishing gradient problem and mode collapse. The second modification is about using the neutrosophic statistical formulation for the 8-connected pixel surrounding the missing pixel. This modification helps to generate a pixel compatible with other surrounding pixels. The second step in the first path is about using the SOM to identify the noisy regions in the satellite images. The third step in the first path is about using the latent diffusion model to remove the noise from the noisy region identified in the previous step. The second path takes the input as tabular satellite data; this path contains only the missing tabulated data using the diffusion model. After the preprocessing steps in both paths, the methodology combines the outputs from the two paths after extracting the regional data from the first path. Then, the methodology uses feature selection to select the features used by the modified LSTM to predict the solar radiation. Fig. 1 shows the methodology diagram.

### The dataset

This study utilizes two datasets: The Solar Irradiation Measurement (SRM) Dataset and the Satellite-based Solar Irradiation (SSR) Dataset. The SRM dataset provides ground-based solar irradiation measurements, while the SSR dataset offers satellite-derived estimates, ensuring a comprehensive approach to solar energy forecasting.

The SRM dataset, compiled by the National Renewable Energy Laboratory (NREL), includes high-precision ground measurements of solar irradiation and related atmospheric variables^45^. The dataset spans the period from January 1, 2010, to December 31, 2020, covering multiple geographical locations across the United States and Europe. The dataset is recorded at an hourly temporal resolution, which ensures enough granularity for capturing variations in solar irradiation and optimizing interpolation and prediction tasks. It comprises twelve essential features, including Global Horizontal Irradiance (GHI), Direct Normal Irradiance (DNI), Diffuse Horizontal Irradiance (DHI), air temperature, relative humidity, wind speed, wind direction, atmospheric pressure, precipitation, and cloud cover. Given the importance of DHI in solar energy applications, this study focuses primarily on this feature to enhance the accuracy of irradiation prediction models. Table 2 provides a detailed description of the key features included in the SRM dataset, highlighting the meteorological and atmospheric parameters that influence solar irradiation levels. These parameters are essential for ensuring accurate model training and validation.

The SRM dataset is primarily derived from the US SURFRAD network, which includes monitoring stations in locations such as Desert Rock, Nevada; Goodwin Creek, Mississippi; and Penn State, Pennsylvania. To ensure data reliability, the dataset undergoes rigorous quality control procedures, including routine validation against known meteorological standards, checks for sensor malfunctions, and removal of anomalous readings. Additionally, nighttime values are excluded to maintain a precise focus on daytime solar irradiation measurements. The dataset is publicly available through the NREL repository, facilitating its use in further research and model development.

**Table 2:** Solar radiation measurement (SRM) dataset.

Feature	Description	Data type
Site location	Latitude, longitude, elevation	Numeric
Timestamp	Date and time	Datetime
Global horizontal irradiance (GHI)	Solar radiation received on a horizontal surface (W/m^2^)	Numeric
Direct normal irradiance (DNI)	Solar radiation received on a surface normal to the sun’s rays (W/m²)	Numeric
Diffuse horizontal irradiance (DHI)	Solar radiation received on a horizontal surface from the sky (excluding the sun) (W/m^2^)	Numeric
Air temperature	Air temperature (°C)	Numeric
Relative humidity	Relative humidity (%)	Numeric
Wind speed	Wind speed (m/s)	Numeric
Wind direction	Wind direction (degrees)	Numeric
Atmospheric pressure	Atmospheric pressure (hPa)	Numeric
Precipitation	Precipitation (mm)	Numeric
Cloud cover	Cloud cover (oktas)	Numeric

The SSR dataset, compiled by the European Space Agency (ESA), consists of satellite imagery and derived solar irradiation products. This dataset provides extensive spatial coverage across Europe and North Africa and includes both tabular data and satellite images in GeoTIFF and JPEG formats. It spans the period from 2010 to 2022, ensuring long-term analysis of solar irradiation trends. The dataset is constructed using multiple satellite sensors, including Sentinel-2, Landsat 8, and MODIS, which offer high-resolution spectral data suitable for solar energy applications^46^. The SSR dataset contains nine essential features, encompassing surface reflectance, cloud cover percentage, aerosol optical depth, water vapor content, surface elevation, and derived solar irradiation parameters such as GHI, DNI, and DHI. In this study, emphasis is placed on the DHI component, as it plays a crucial role in improving solar irradiation predictions. Table 3 presents a comprehensive overview of the features included in the SSR dataset, detailing the satellite-based parameters utilized in this study.

**Table 3:** Satellite-based solar radiation (SSR) dataset.

Feature	Description	Data type
Satellite sensor	Satellite sensor (e.g., Sentinel-2, Landsat 8, MODIS)	Categorical
Timestamp	Date and time	Datetime
Geographical location	Latitude, longitude	Numeric
Surface reflectance	Visible, near-infrared, shortwave infrared band reflectance	Numeric
Cloud cover percentage	Percentage of cloud cover	Numeric
Aerosol optical depth	Measure of aerosol concentration in the atmosphere	Numeric
Water vapor content	Water vapor content in the atmosphere (g/m^2^)	Numeric
Surface elevation	Elevation of the surface (m)	Numeric
Derived solar radiation	Estimated GHI, DNI, DHI (W/m^2^)	Numeric
Satellite imagery	Satellite images capturing the atmospheric and surface conditions	Image

The selection of these datasets is based on multiple criteria, including spatial resolution, temporal coverage, and data reliability. The SRM dataset offers high temporal accuracy, making it an ideal reference for validating satellite-derived predictions. In contrast, the SSR dataset provides superior spatial granularity, which is critical for capturing localized atmospheric variations. While geostationary satellites offer higher temporal resolution, their spatial coverage is often less detailed compared to Low Earth Orbit (LEO) satellites, such as those utilized in this study. The advantage of LEO-based datasets lies in their ability to provide fine-scale spatial details, though they are limited by longer revisit times, which can affect real-time applications.

By integrating ground-based and satellite-derived datasets, this study ensures greater accuracy and robustness in solar irradiation modeling. The SRM dataset provides high-reliability data across North America and Europe, whereas the SSR dataset extends the applicability of the model to regions with limited ground-based data coverage, such as North Africa. This combination establishes a comprehensive foundation for developing scalable and precise solar irradiation forecasting models. Both datasets are publicly accessible, with the SRM dataset available via the NREL repository and the SSR dataset accessible through the ESA Earth Observation Data Portal.

### Pixel imputation using hybrid random forest and GANs with 8-connected pixel analysis

This part of the methodology used to impute the missing pixels of the satellite images. The missing pixel may occur during transmission and capturing. This part of the methodology contains combination of multiple parts as mentioned in figure. The hybrid model in the block diagram contains two main parts. The first part is the random forest model. The output value of the random forest algorithm is used as the input value of the generative adversarial interpolation network. And the second part is the GANs with identity block and 8-connected pixels. This part of the methodology contains set of novel modification; the first modification is about to add the identity block; the identity block is to solve the mode collapse problem and the vanishing gradient problem. This help network to generate different pixel each time of training. The second modification is about using the 8-connected pixel which used to calculate the average value of the surrounding pixels using neutrosophic statistical analysis method. The neutrosophic used to give value of each 8 connected pixel and then calculate the average value of the 8-connected pixels. The GANs network generate value of the missing pixel and after generation the pixel; it compared with the value of the average of the 8-connected pixel. The model stops when the generated value is near the average the 8-connected pixel.

**Fig. 2:**
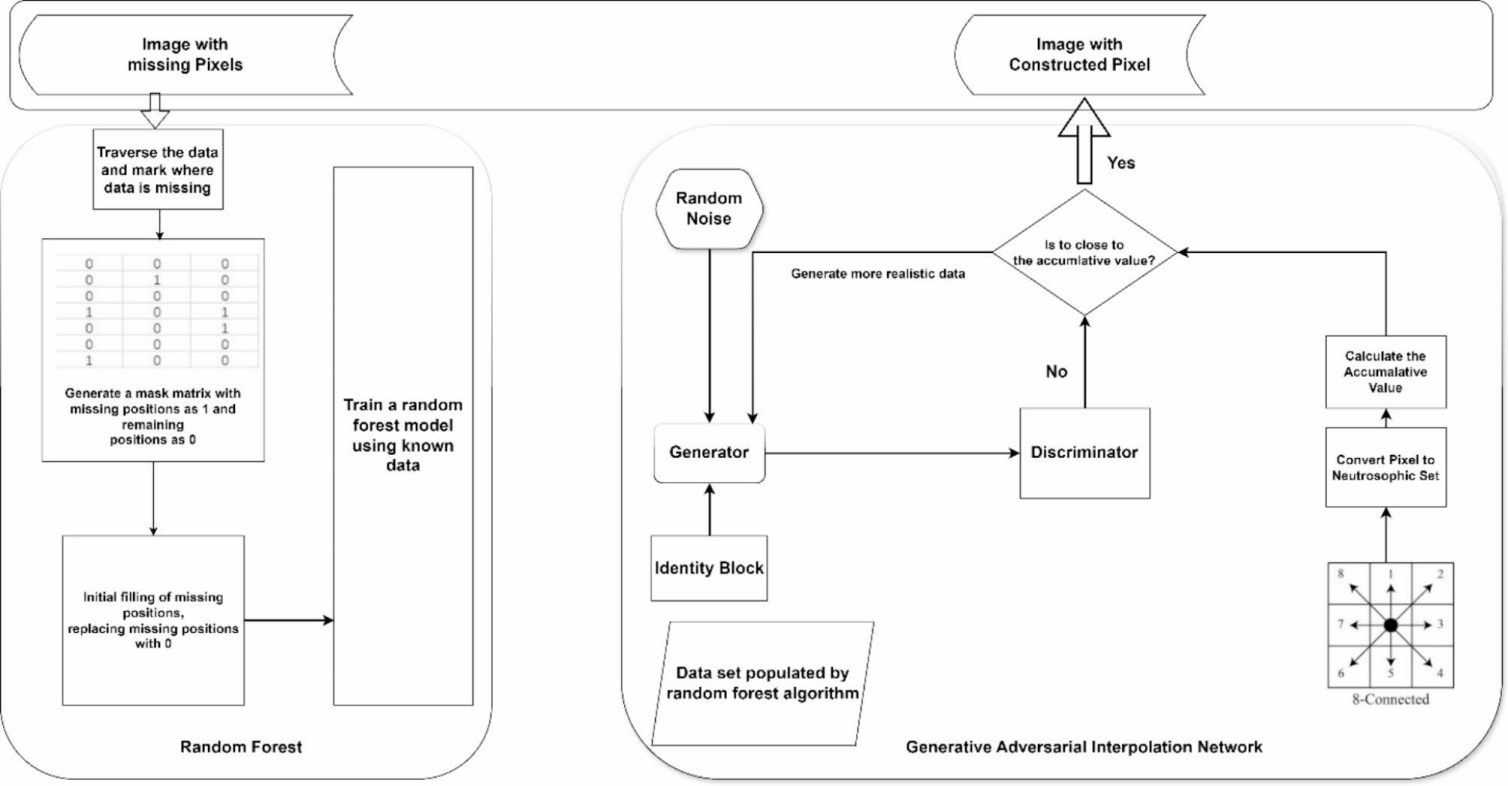
Block diagram of random forest with identity GANs.

The process begins with the application of Random Forest, which is used to predict the missing pixel values based on the available surrounding pixel information. The RF model is particularly effective in capturing the complex dependencies within the data due to its ensemble learning nature. However, Random Forest alone may not fully account for the intricate patterns and variability present in satellite images, which is where the integration of GANs becomes essential as illustrated in Fig. 2.

**Fig. 3:**
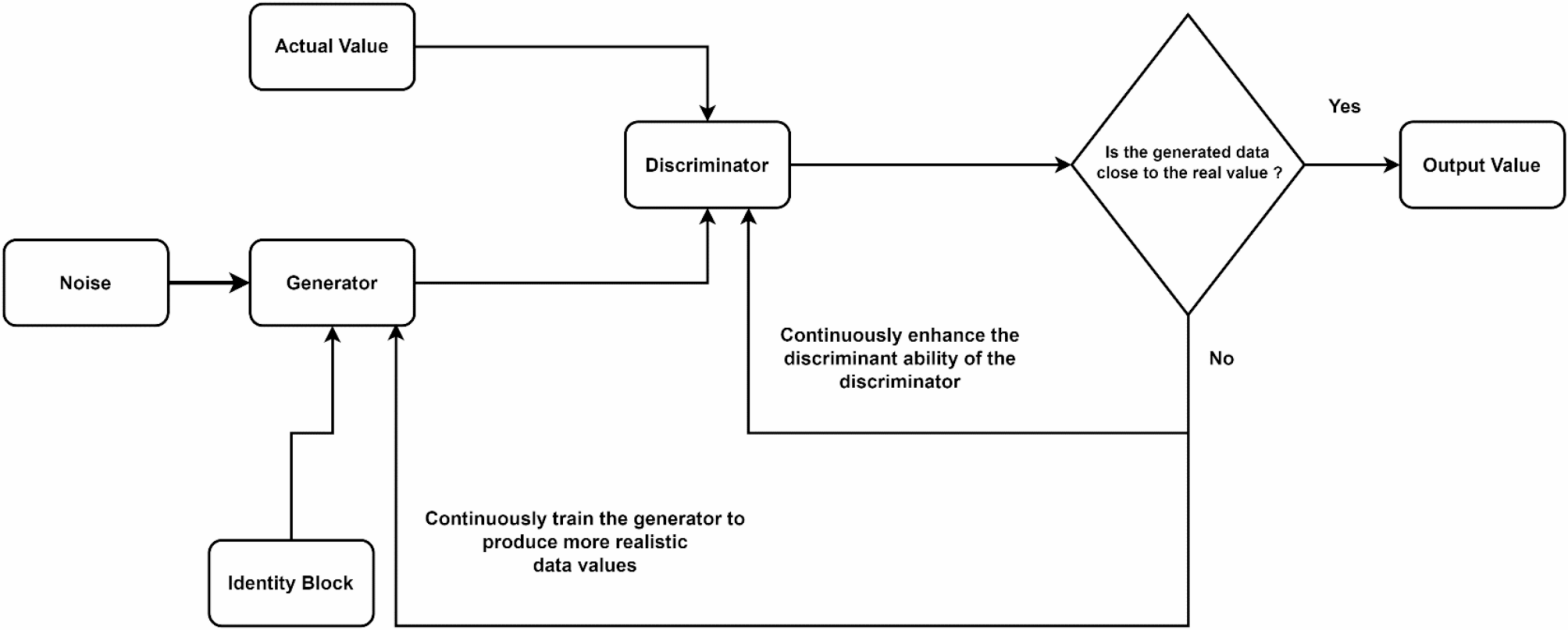
Basic framework of generative adversarial network.

Generative Adversarial Networks (GANs), as depicted in Fig. 3, are then utilized to refine the imputed pixel values generated by the RF model. The GAN architecture comprises two main components: a generator and a discriminator. The generator attempts to create realistic pixel values, while the discriminator evaluates the authenticity of these generated values against the real data. This adversarial process continues until the generator produces pixel values that are indistinguishable from the actual data, thereby enhancing the quality of the imputation.

**Fig. 4:**
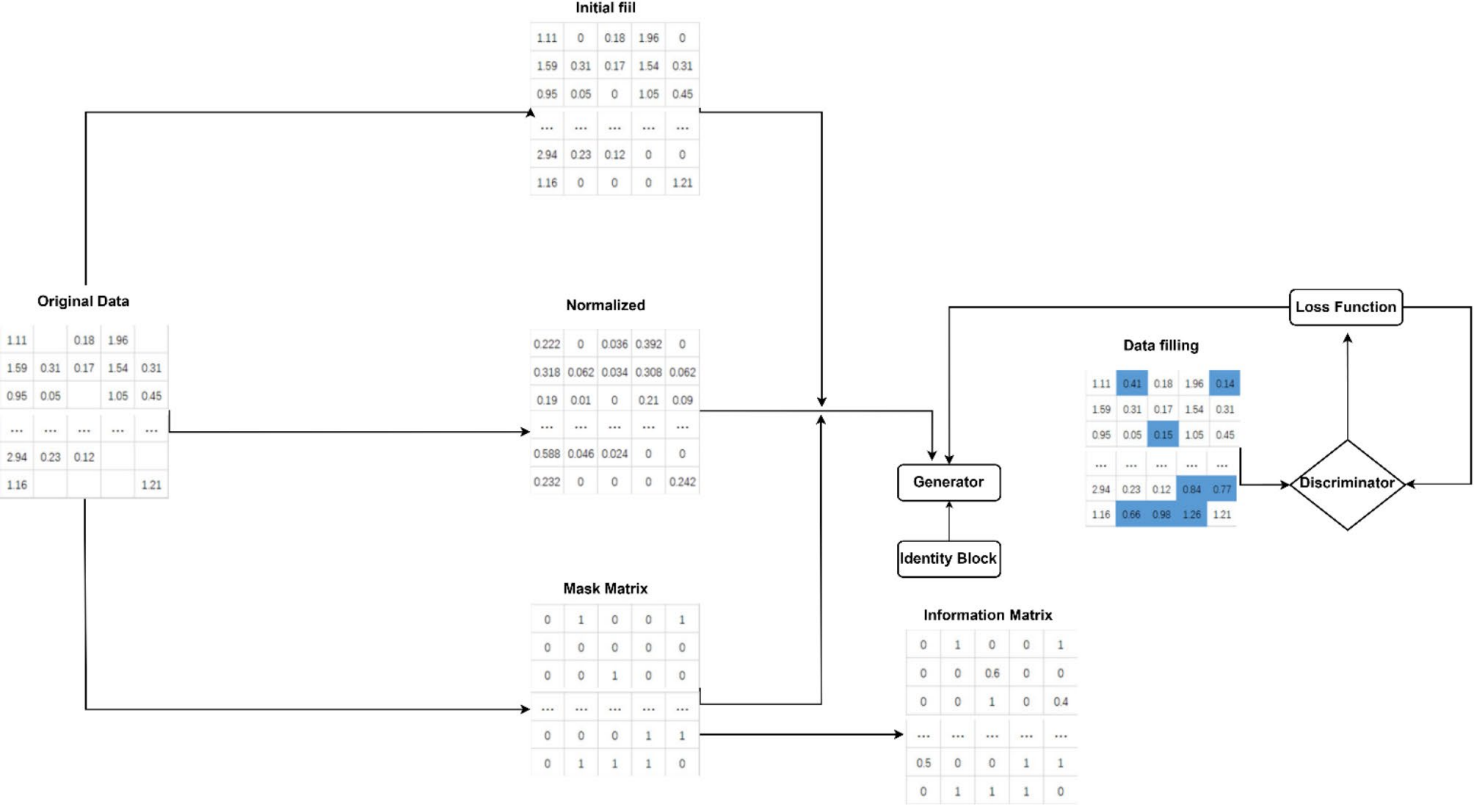
Generative adversarial interpolation network (GAIN) framework.

To further refine the imputation, the framework incorporates an 8-connected pixel analysis, a technique that considers the spatial relationships between a pixel and its eight immediate neighbors. This analysis ensures that the imputed pixels are not only accurate in isolation but also consistent with the surrounding pixel structure, preserving the overall integrity of the satellite images. As depicted in Fig. 4, the final step involves the integration of the outputs from both the RF model and the GANs, resulting in a robust imputation model capable of handling the complexities of satellite image data. The combined approach of Random Forest and GANs, supported by 8-connected pixel analysis, provides a significant improvement in the prediction of solar energy radiation, ensuring that the imputed data is both accurate and reliable.

### Noisy regions identification with SOM

The Time Series Growing Self-Organizing Map (TS-GSOM) is a powerful technique that can be effectively employed to identify and remove noise from satellite imagery. The TS-GSOM is an unsupervised neural network model that can learn the underlying patterns and structures within a time series of satellite images. By treating each pixel in the satellite images as a time series, the TS-GSOM can capture the spatial and temporal characteristics of the data, enabling it to distinguish between genuine features and noise. The model begins with a small initial map and adaptively grows its size and complexity as it learns the intricate patterns present in the satellite data. This dynamic growth allows the TS-GSOM to identify regions within the images that exhibit anomalous or noisy behavior, which can then be selectively targeted for further denoising. The unsupervised nature of the TS-GSOM makes it particularly well-suited for satellite imagery, where the sources and characteristics of noise can be highly complex and variable. By leveraging the TS-GSOM’s ability to learn the data’s inherent structure, satellite image analysts can effectively isolate and remove noise, leading to more accurate and reliable interpretation of the underlying land cover, environmental changes, and other important geospatial information.

The TS-GSOM model consists of three key components: the input layer, the growing self-organizing map, and the output layer. The input layer takes in the time series of satellite image pixels, treating each pixel as a multivariate time series. The growing self-organizing map forms the core of the model, starting with a small initial grid of neurons and adaptively expanding its size and complexity as it learns the underlying patterns in the data. The learning process involves competitive learning, where neurons compete to represent the input data, and cooperative learning, where neighboring neurons adjust their weights to capture the spatial and temporal relationships. As the map grows, it forms clusters of neurons that correspond to distinct features and structures within the satellite images, allowing the model to differentiate between genuine image content and noise. Finally, the output layer aggregates the learned representations from the growing self-organizing map, providing a denoised version of the input satellite images by selectively reconstructing the non-noisy regions. The TS-GSOM model operates in an iterative fashion, with each iteration refining the map and improving the noise removal capabilities. By leveraging the self-organizing and adaptive nature of the growing map, the TS-GSOM can effectively identify and suppress the noise in satellite imagery, leading to enhanced image quality and more accurate interpretation of the underlying geospatial information as shown in Pseudocode (3 -1).

**Figure:**
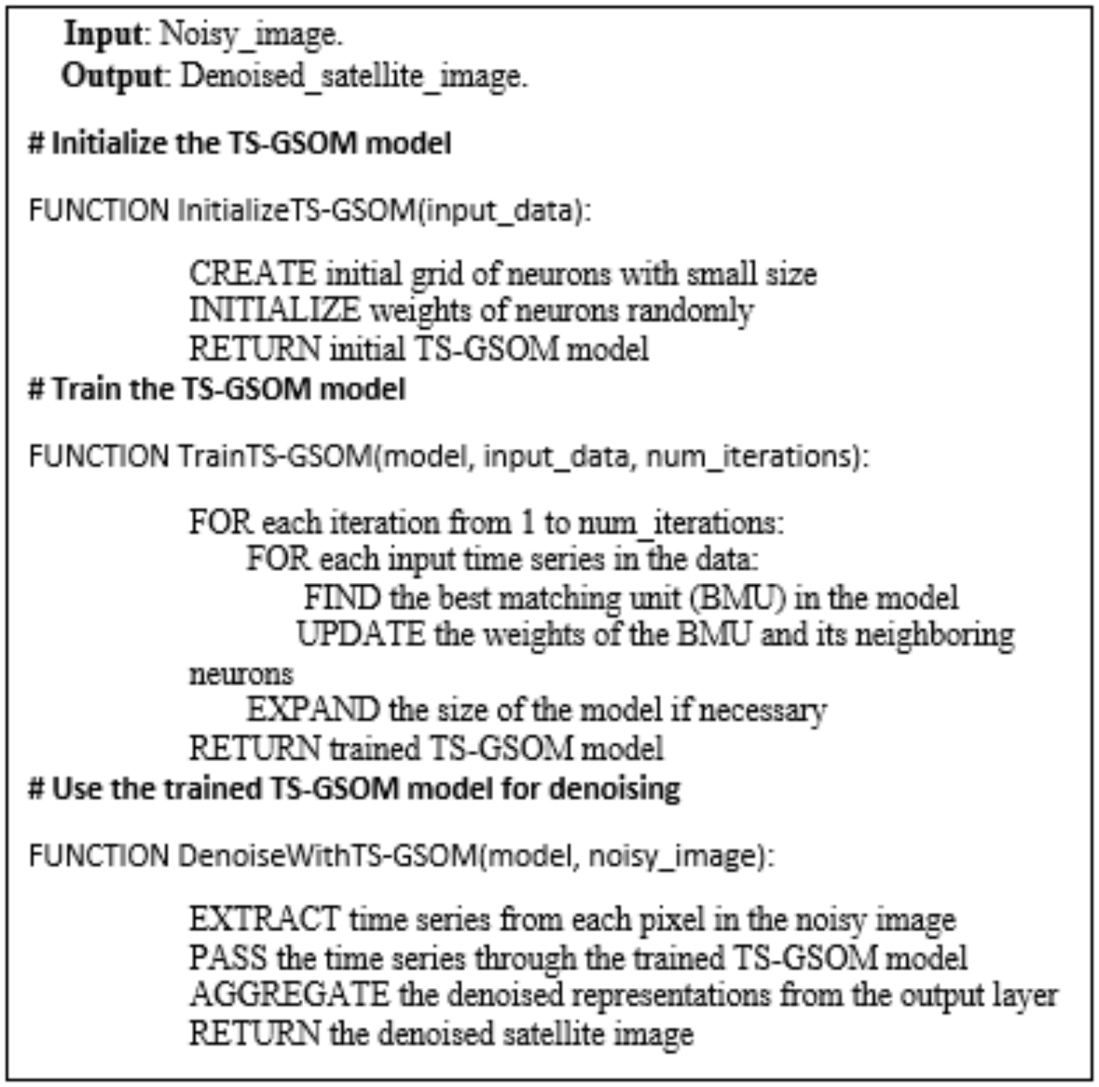
Pseudocode (3-1): The TS-GSOM model.

### Noise removal using the latent diffusion model

The use of a latent diffusion model is a promising approach for removing noise from the satellite imagery. Latent diffusion models are a type of generative neural network that can effectively map noisy input images to a clean, noise-free representation in a learned latent space. By training the latent diffusion model on a dataset of high-quality satellite images, the model can learn the underlying patterns and features that characterize clear, unobstructed imagery., the trained model can then iteratively denoise the input, progressively removing unwanted artifacts and distortions while preserving the important structural and spectral information. This allows for the recovery of a clean, high-fidelity representation of the ground features, which is crucial for accurately measuring and monitoring the archaeological site over time.

This latent space encodes the essential features of the image in a lower-dimensional form, while filtering out the unwanted noise. The decoder then takes this clean, noise-free latent representation and generates a reconstructed output image that closely matches the original, high-quality version. The training of the latent diffusion model involves iteratively refining this encoding-decoding process, minimizing the reconstruction error between the model output and the ground truth clean images. This allows the network to learn an effective mapping from the noisy input to the denoised output, enabling it to generalize and denoise new satellite images with high fidelity. The encoder and decoder architecture, along with the diffusion-based training process, are the core components that give the latent diffusion model its powerful denoising capabilities for satellite imagery.

The latent diffusion network operates by progressively transforming a noisy input satellite image into a clean, denoised output. This is achieved through a series of diffusion steps, where the network gradually removes the noise while preserving the underlying image features. The process begins with the encoder, which takes the noisy input image and maps it to a compact latent representation. This latent encoding captures the essential image information in a lower-dimensional form, effectively separating the signal from the noise. The decoder then uses this clean latent representation to generate the denoised output image, restoring the visual quality and details. Crucially, the training of the latent diffusion model involves iteratively adding and removing noise from the input images, learning the inverse mapping that can effectively denoise new samples. By modeling this diffusion process, the network develops a robust understanding of how to remove unwanted artifacts and distortions from the satellite imagery, while maintaining the important structural and spectral characteristics. Through this iterative, diffusion-based approach, the latent diffusion model can produce high-quality, denoised satellite images that are crucial for accurate analysis and interpretation of the target site as shown in Psuedocode (3-2).

**Figure:**
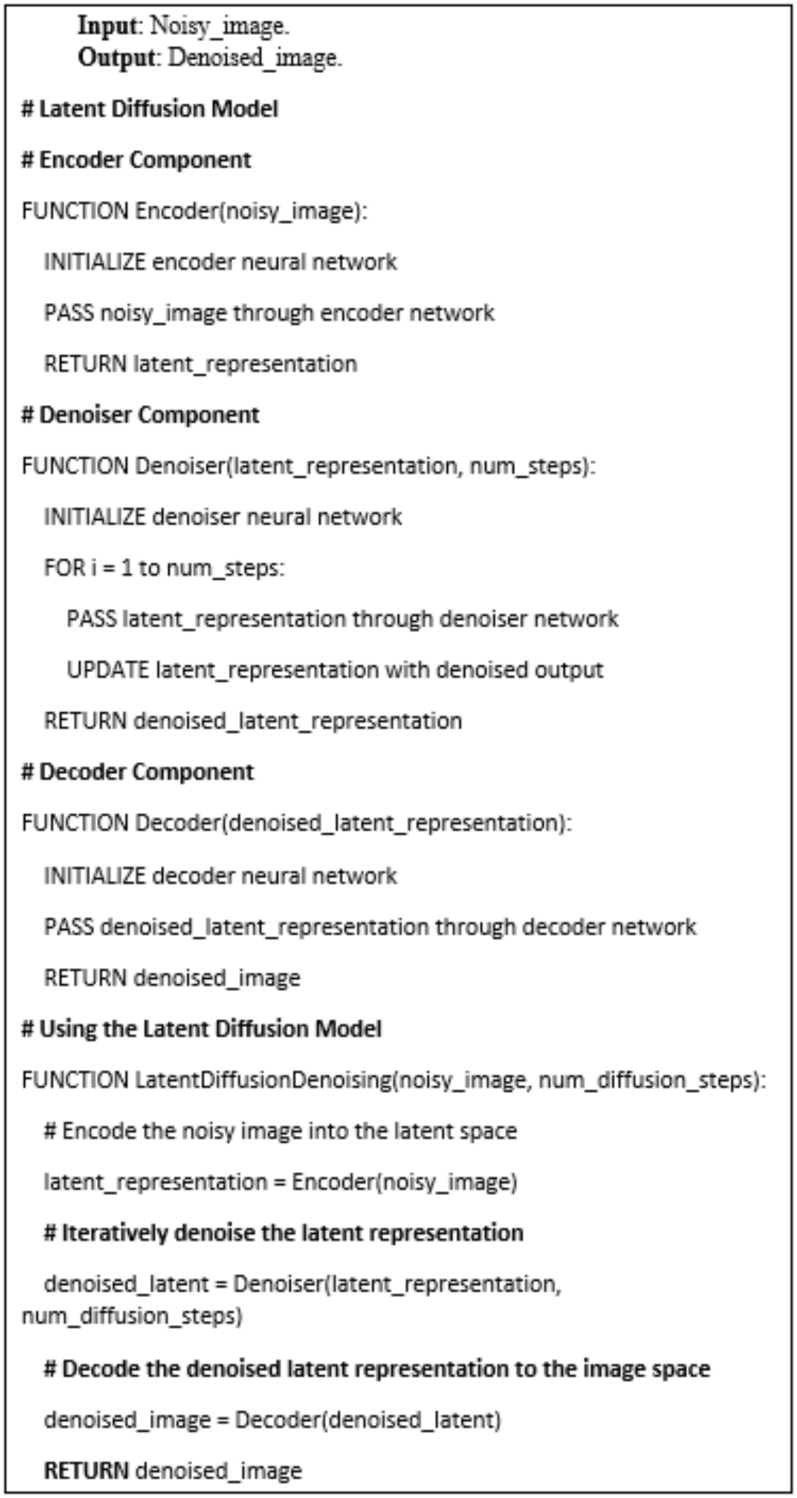
Pseudocode (3-2): The latent diffusion model components.

### Missing data imputation using diffusion model

To handle the issue of missing values in the satellite-derived tabular data, we employed a diffusion-based imputation approach. Due to factors such as sensor malfunctions, atmospheric interference, or insufficient ground coverage, some entries in the tabular dataset derived from the satellite imagery were missing. We addressed this by modeling the underlying data distribution using a diffusion probabilistic model. Specifically, we trained a conditional diffusion model that could generate plausible completions for the missing entries based on the observed non-missing features. The diffusion model learned the complex statistical relationships between the different variables in the tabular data by iteratively adding controlled noise and then reversing the process to recover the original data distribution. Once trained, we used the diffusion model to sample likely values for the missing entries, conditioning on the known feature values for each row. This approach allowed us to impute the missing data in a manner that preserved the multivariate structure and higher-order statistics of the original satellite-derived tabular dataset. The diffusion-based imputation provided more accurate and realistic estimates compared to simpler techniques, enabling us to maximize the information content used in the subsequent data analysis.

The diffusion-based imputation approach we employed consisted of several key components. At the core was a conditional diffusion probabilistic model that learned the underlying data distribution of the satellite-derived tabular dataset. This diffusion model was composed of a noise prediction neural network and a Markov Chain Monte Carlo (MCMC) sampling procedure. The noise prediction network took as input the known feature values for a row with missing entries, and outputted predictions of the noise that would need to be sequentially added to generate the missing values. The MCMC sampling then iteratively applied this learned noise addition process in reverse, starting from random initializations, to produce plausible completions for the missing entries that matched the observed data distribution.

The key steps of missing tabular data imputation as the follow:

1. Train a noise prediction model on the complete data.
2. For each row with missing values:
  - Randomly initialize the missing values.
  - Perform MCMC sampling to update the missing values:
    (i) Predict the noise to add using the trained model.
    (ii) Update the missing values by adding the predicted noise.
    (iii) Accept the new values based on an MCMC criterion.
  - Update the original row with the final imputed values.
3. Return the dataset with the imputed missing values.

And pseudocode (3-3) and (3-4) refers to how the missing data imputation using the diffusion model as the follow.

**Figure:**
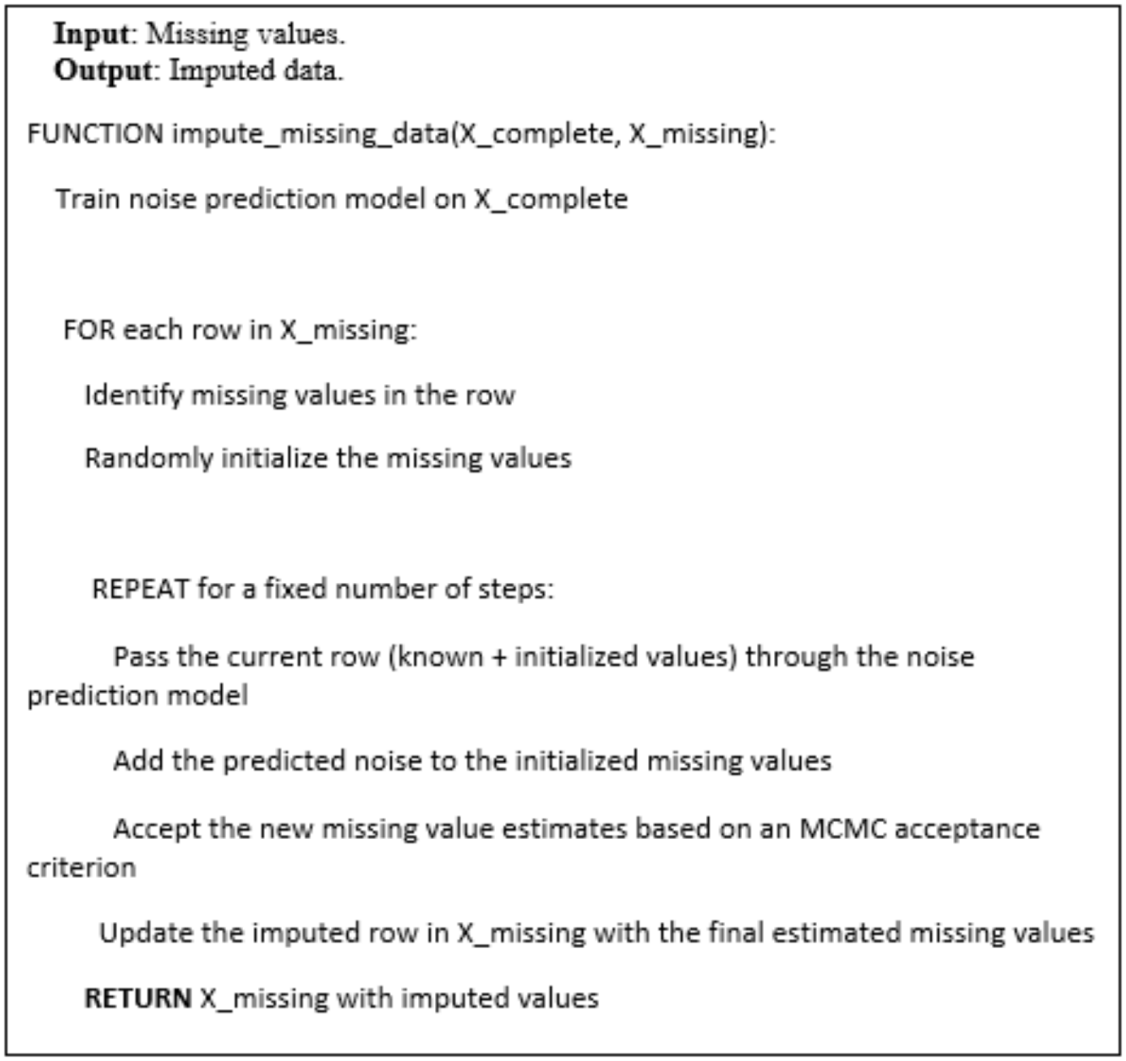
Pseudocode (3-3): The diffusion-based imputation process.

**Figure:**
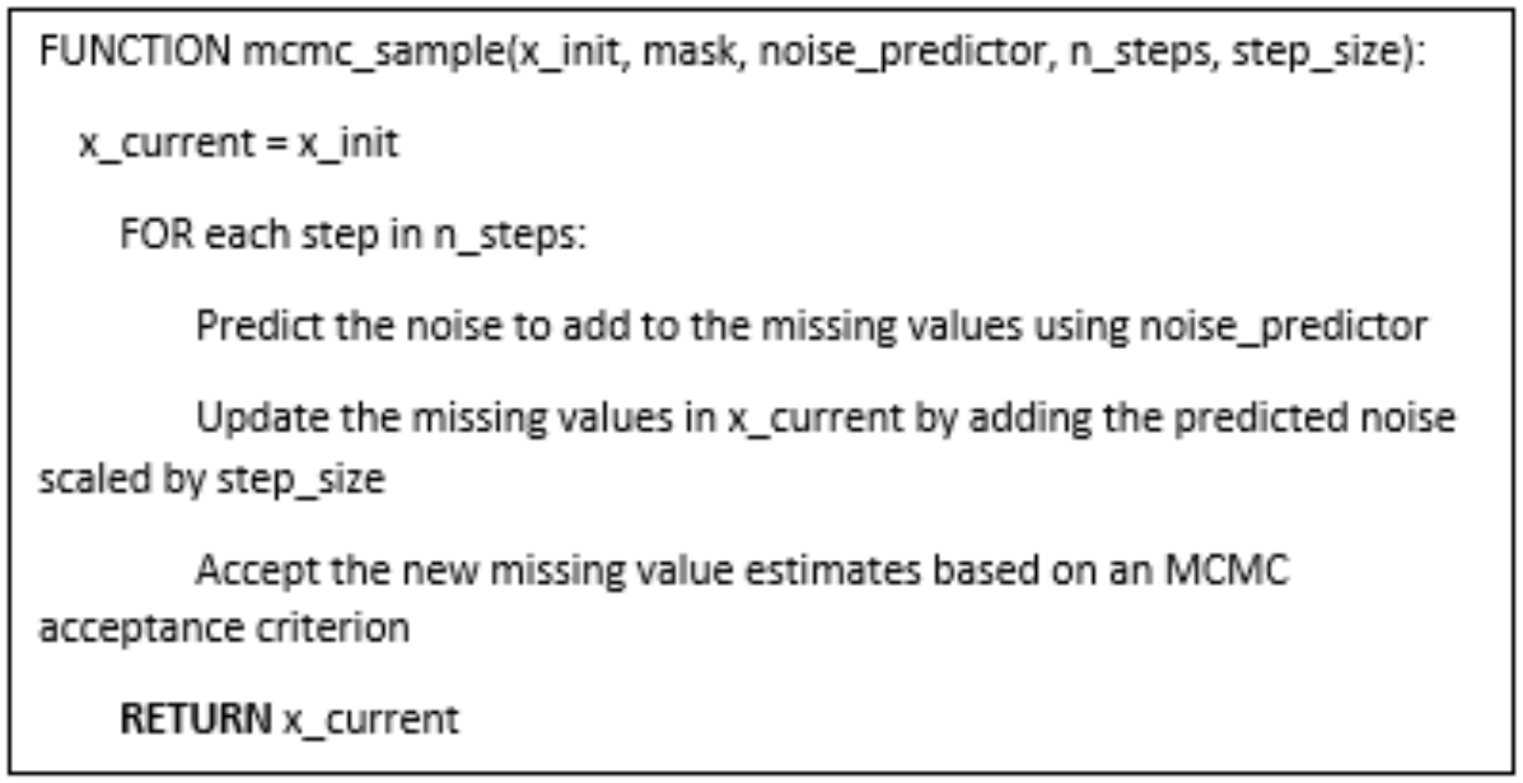
Pseudocode (3-4) : MCMC sampling step.

### Features extraction

In this study, feature extraction was performed by integrating information from two distinct datasets to construct a comprehensive set of predictors for the LSTM forecasting model. The first dataset consisted of tabular data containing financial, economic, and demographic variables, while the second dataset provided geographical information derived from satellite imagery, including land cover classifications, vegetation indices, and infrastructure characteristics. To ensure a seamless integration of these datasets, a spatial join operation was applied, aligning the tabular data with their corresponding geographic coordinates. This process enriched each tabular record with relevant satellite-derived features corresponding to specific locations.

Following data integration, feature engineering techniques were employed to capture complex relationships between the tabular and geospatial attributes. The engineered features incorporated spatial lags, geographic clustering metrics, and multimodal representations of satellite imagery, enhancing the predictive capacity of the model. To refine the feature set, a rigorous selection process was conducted using recursive feature elimination (RFE) and permutation importance. This procedure identified the most informative predictors, which primarily included global horizontal irradiance, direct normal irradiance, and air temperature from the SRM dataset. Additionally, surface reflectance in the visible and near-infrared bands, aerosol optical depth, and cloud cover percentage from the SSR dataset demonstrated significant contributions to the predictive model. These features exhibited strong importance scores and effectively captured the spatial and temporal variations in solar radiation.

To address potential multicollinearity among the selected features, the Variance Inflation Factor (VIF) was computed for each variable. Features with high collinearity, indicated by a VIF exceeding a predefined threshold, were either removed or transformed using Principal Component Analysis (PCA). This approach ensured that the final feature set retained its predictive capacity while minimizing redundancy. By leveraging a diverse set of predictors from both tabular and geospatial sources and applying systematic feature selection and dimensionality reduction techniques, the constructed feature set was optimized to enhance the accuracy and reliability of solar irradiation predictions. Table 4 provides a summary of the selected features and their corresponding importance scores, highlighting their respective contributions to the model’s predictive performance.

**Table 4:** Selected features and their importance scores.

Feature	Importance score	Dataset
Global horizontal irradiance (GHI)	0.95	SRM
Direct normal irradiance (DNI)	0.92	SRM
Air temperature	0.89	SRM
Surface reflectance (visible band)	0.87	SSR
Aerosol optical depth	0.85	SSR
Cloud cover percentage	0.83	SSR

### Solar radiation prediction using modified LSTM (LSTM with seasonality and trend handling)

The traditional LSTM model, while effective at capturing complex temporal patterns, may struggle to accurately predict solar radiation due to the strong seasonal and trend components inherent in such time series data. To address this limitation, the Modified LSTM model incorporates dedicated mechanisms to explicitly handle the seasonality and trend present in the input solar radiation data. Specifically, the input time series is first decomposed into its seasonal, trend, and residual components using techniques such as Seasonal-Trend decomposition using Loess (STL). The seasonal and trend components are then fed into separate LSTM sub-networks, allowing the model to learn the unique characteristics of these various data features. The outputs of the seasonal and trend LSTM sub-networks are then combined with the residual component to produce the final solar radiation prediction. This explicit modeling of the underlying drivers of solar radiation helps the Modified LSTM overcome the limitations of the traditional LSTM, resulting in improved forecasting accuracy.

The Modified LSTM model for solar radiation prediction is designed with several key components. It begins with an input layer that processes the solar radiation time series. This input is then passed through a decomposition module that divides the time series into seasonal, trend, and residual components. The seasonal and trend components are each processed by separate LSTM sub-networks, which have their own LSTM units and internal states. These sub-network outputs are then concatenated with the residual component and passed through a final dense layer to generate the solar radiation prediction. This architecture enables the model to capture the unique characteristics of the seasonal, trend, and residual components, resulting in more accurate forecasts compared to the traditional LSTM approach, as demonstrated by the hyperparameters detailed in Table 5.

**Table 5:** Hyperparameters of LSTM approach.

Hyperparameter	Value
Number of LSTM units in seasonal sub-network	128
Number of LSTM units in trend sub-network	64
Learning rate	0.0001
Batch size	64
Number of training epochs	200 epochs with three stopping criteria

## Results

This section presents the results obtained from the proposed methodology, highlighting its effectiveness in solar radiation forecasting. The proposed model is a forecasting model, as it uses historical data (e.g., past satellite images and meteorological data) to predict future solar radiation values. The results demonstrate the effectiveness of our forecasting model in predicting future solar radiation values with high accuracy. The evaluation is structured into two main parts: accuracy metrics and performance evaluation. The accuracy metrics provide a comprehensive assessment of the predictive capabilities of the proposed model by analyzing key evaluation measures such as accuracy, precision, recall, and F1-score. This analysis ensures a detailed understanding of the model’s ability to generate reliable predictions. The performance evaluation examines the effectiveness of the proposed approach based on the obtained results. This analysis includes a comparative assessment against baseline models, providing insights into the strengths and limitations of the methodology. The evaluation further explores the impact of the preprocessing steps and feature selection on the final prediction accuracy. Through this structured assessment, the results validate the robustness of the proposed deep learning framework in integrating satellite images and tabular data for solar radiation forecasting.

### Accuracy metrics

The performance of the proposed solar radiation prediction model was evaluated using a comprehensive set of well-established metrics. These evaluation measures provide a detailed assessment of the model’s accuracy, reliability, and effectiveness in forecasting solar radiation. By analyzing these quantitative indicators, the study ensures a rigorous examination of the model’s predictive capabilities and its ability to generate precise and dependable forecasts. Since the model operates at an hourly resolution, all evaluation metrics were calculated based on hourly predictions to ensure a precise assessment of performance at this temporal scale.

The Mean Absolute Error (MAE), defined in Eq. (1), was utilized to assess the average absolute difference between the predicted solar radiation values ($\:{G}_{i}$) and the ground truth observations ($\:G{P}_{i}$). MAE offers a clear indication of the typical magnitude of the errors, with lower values indicating superior model performance.

1 $$\:MAE=\frac{1}{n}\sum\:_{i=1}^{n}|{G}_{i}-G{P}_{i}|$$

Furthermore, the Mean Squared Error (MSE), presented in Eq. (2), was calculated to measure the average squared difference between the predicted and actual solar radiation values. MSE is sensitive to large errors, making it a valuable metric for identifying and addressing significant discrepancies in the model’s forecasts.

2 $$\:MSE=\frac{1}{n}\sum\:_{i=1}^{n}n{({G}_{i}-G{P}_{i})}^{2}$$

Building upon MSE, the Root Mean Squared Error (RMSE), as shown in Eq. (3), was employed to provide a measure of the average magnitude of the errors in the same units as the original data. RMSE offers an intuitive interpretation of the model’s performance within the context of the solar radiation prediction problem.

3 $$\:RMSE=\sqrt{\frac{1}{n}\sum\:_{i=1}^{n}n{({G}_{i}-G{P}_{i})}^{2}}$$

The Coefficient of Determination, or R-Squared (R^2^), calculated using Eq. (4), was utilized to assess the proportion of the variance in the dependent variable (solar radiation) that is predictable from the independent variables. R^2^ values range from 0 to 1, with higher values indicating a better model fit.

4 $$\:{R}_{\text{Squared\:}}=1-\frac{S{S}_{\text{regression\:}}}{S{S}_{\text{total\:}}}\:$$

To evaluate the quality and diversity of the generated samples, the Inception Score (IS), defined in Eq. (5), was employed. IS measures the exponentiation of the expected value of the Kullback-Leibler (KL) divergence between the conditional class probability distribution and the marginal class probability distribution, with higher values indicating better-quality and more diverse generated samples.

5 $$\:IS=\text{e}\text{x}\text{p}\left({E}_{x\sim\:{p}_{g}}{D}_{KL}\right(p(y\mid\:x)\Vert\:p\left(y\right)\left)\right)$$

Additionally, the Fréchet Inception Distance (FID), calculated using Eq. (6), was utilized to assess the similarity between the generated samples and the real samples by comparing the mean and covariance of the feature vectors extracted from the Inception model. Lower FID values indicate better-quality and more realistic generated samples.

6 $$\:\text{FID\:}={\Vert\:{\mu\:}_{r}-{\mu\:}_{g}\Vert\:}^{2}+\text{T}\text{r}({\text{S}}_{r}+{\text{S}}_{g}-2\sqrt{\left({\text{S}}_{r\:}{\text{S}}_{g}\right)})$$

The Structural Similarity Index (SSIM), defined in Eq. (7), was employed to evaluate the structural similarity between the generated samples and the real samples, considering luminance, contrast, and structural information. SSIM values range from 0 to 1, with higher values indicating better structural similarity.

7 $$\:SSIM=\sqrt{\frac{1}{N}\times\:\sum\:_{n=1}^{N}n{({x}_{i}-\hat {{x}}_{i})}^{2}}$$

Finally, the Peak Signal-to-Noise Ratio (PSNR), calculated using Eq. (8), was utilized to measure the ratio between the maximum possible signal value and the noise level, providing a measure of the image quality. Higher PSNR values indicate better image quality.

8 $$\:PSNR=\sqrt{\frac{\sum\:_{n=1}^{N}n{({x}_{i}-\hat {{x}}_{i})}^{2}}{\sum\:_{n=1}^{N}n{x}_{i}^{2}}}\times\:100$$

The employment of these diverse evaluation metrics provides a comprehensive assessment of the proposed solar radiation prediction model’s accuracy, reliability, and overall effectiveness. By leveraging this suite of quantitative measures, the researchers can thoroughly evaluate the model’s performance and identify areas for further improvement, ultimately enhancing the reliability and applicability of the solar radiation forecasting system.

### Performance evaluation

**Fig. 5:**
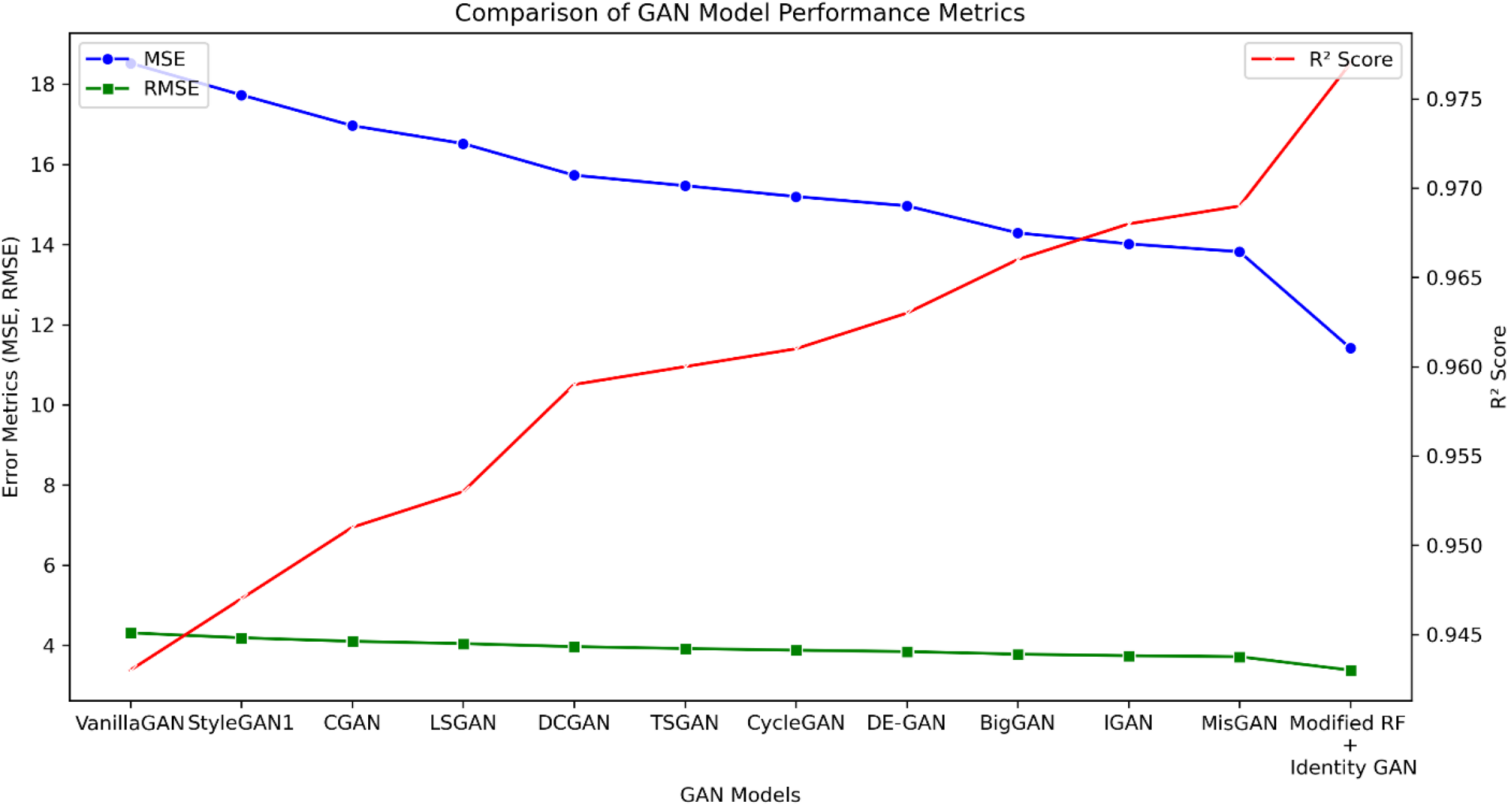
Comparison between modified RF and identity GAN and other GAN models in missing pixel imputation.

This part of the paper introduces the results of the different sections of the methodology, including pixel and data imputation, noise removal, and data predictions. This section also presents a comparison between our model and other models performing the same task. Table 6 presents the results of pixel imputation for satellite images, using eight different metrics to evaluate the effectiveness of the imputation task. The experimental results demonstrate the superior performance of the Modified RF + Identity GAN compared to other models in pixel imputation. The Modified RF + Identity GAN achieves the best performance across most metrics, with a Mean Squared Error (MSE) of 11.412 W^2^/m^4^, Root Mean Squared Error (RMSE) of 3.382 W/m^2^, and R-squared (R^2^) of 0.977, showcasing its robustness in reconstructing missing pixel data. As illustrated in Fig. 5, the Modified RF + Identity GAN consistently outperforms other models across key error metrics, achieving the lowest MSE and RMSE while maintaining the highest R^2^ value. Other models, such as MisGAN, IGAN, and BigGAN, also demonstrate competitive performance, with MisGAN achieving an MSE of 13.819 W^2^/m^4^, RMSE of 3.717 W/m^2^, and R^2^ of 0.969. Figure 5 provides a visual comparison of these models using line plots, allowing for an intuitive interpretation of the performance trends across different evaluation metrics.

**Table 6:** Comparison of pixel imputation for various GAN architectures.

GAN model	MSE (W^2^/m^4^)	RMSE (W/m^2^)	MAE (W/m^2^)	*R*²	MAPE (%)	FID	IS
VanillaGAN^47^	18.522	4.312	2.519	0.943	17.498	27.571	3.26
StyleGAN1^48^	17.731	4.189	2.354	0.947	16.584	25.245	3.31
CGAN^49^	16.965	4.102	2.319	0.951	16.052	24.157	3.37
LSGAN^50^	16.518	4.045	2.348	0.953	15.919	23.642	3.38
DCGAN^51^	15.728	3.968	2.185	0.959	15.174	22.645	3.46
TSGAN^52^	15.467	3.921	2.122	0.960	15.049	22.159	3.44
CycleGAN^53^	15.196	3.879	2.183	0.961	15.276	22.319	3.48
DE-GAN^54^	14.965	3.845	2.094	0.963	14.694	21.571	3.50
BigGAN^55^	14.289	3.781	2.078	0.966	14.468	21.319	3.56
IGAN^56^	14.015	3.742	2.031	0.968	14.045	20.892	3.59
MisGAN^57^	13.819	3.717	2.018	0.969	13.975	19.895	3.66
Modified RF + identity GAN	11.412	3.382	1.901	0.977	11.612	17.215	3.81

**Fig. 6:**
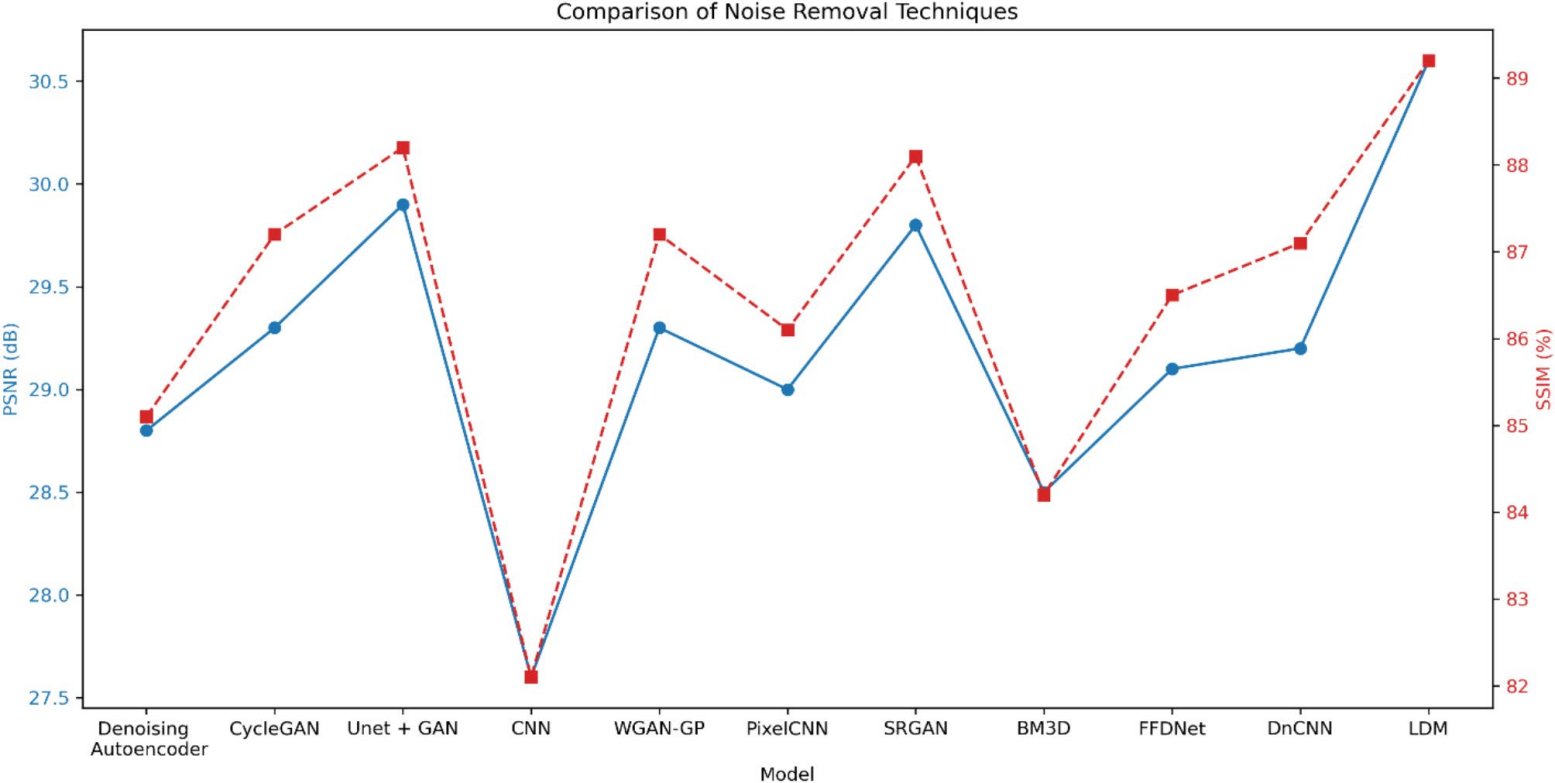
Comparison between LDM and other models in removing noise.

The comparative performance of various image denoising models was evaluated and is presented in Table 7. The models were assessed using two key evaluation metrics: Peak Signal-to-Noise Ratio (PSNR) in decibels (dB) and Structural Similarity Index (SSIM). The analysis revealed that the Latent Diffusion Model (LDM) outperformed all other techniques in both PSNR and SSIM. The LDM achieved a PSNR of 30.6 dB and an SSIM of 0.892, indicating its superior ability to preserve the structural and perceptual quality of the denoised images. As illustrated in Fig. 6, the PSNR and SSIM trends clearly highlight the superiority of LDM over the other techniques. The SRGAN (Super-Resolution GAN) and Unet + GAN models also demonstrated strong performance, with PSNR values of 29.8 dB and 29.9 dB, respectively, and SSIM values of 0.881 and 0.882, respectively. This suggests that the combination of generative adversarial networks and convolutional neural networks can be highly effective in image denoising tasks. The WGAN-GP (Wasserstein GAN with Gradient Penalty) and DnCNN (Denoising Convolutional Neural Network) models followed closely, with PSNR values around 29.2 dB and SSIM values of 0.871. Other models, such as the Denoising Autoencoder, PixelCNN, and FFDNet, performed moderately well, while the Convolutional Neural Network (CNN) and BM3D (Block-Matching and 3D Filtering) exhibited the lowest performance, with PSNR values of 27.6 dB and 28.5 dB, and SSIM values of 0.821 and 0.842, respectively. These results, visualized in Fig. 6, reinforce the effectiveness of LDM in achieving higher fidelity in noise removal compared to conventional and GAN-based approaches.

**Table 7:** Comparison of noise removal techniques.

Model	PSNR (dB)	SSIM (%)
Denoising autoencoder^58^	28.8	85.1
CycleGAN^59^	29.3	87.2
Unet + GAN^60^	29.9	88.2
Convolutional neural network (CNN)^38^	27.6	82.1
WGAN-GP (Wasserstein GAN with gradient penalty)^61^	29.3	87.2
PixelCNN^62^	29.0	86.1
SRGAN (super-resolution GAN)^63^	29.8	88.1
BM3D (block-matching and 3D filtering)^64^	28.5	84.2
FFDNet (fast and flexible denoising network)^65^	29.1	86.5
DnCNN (denoising convolutional neural network)^66^	29.2	87.1
Latent diffusion model (LDM)	30.6	89.2

**Fig. 7:**
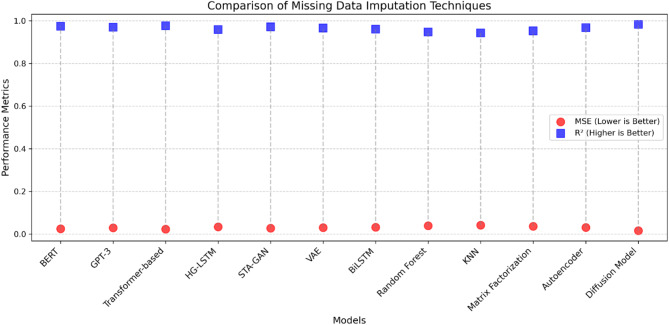
Comparison between diffusion model and other model in data imputation.

The performance of various image denoising models was rigorously evaluated, and the results are presented in Table 8. The assessment was conducted using two key metrics: Peak Signal-to-Noise Ratio (PSNR) and Structural Similarity Index (SSIM). The standout performer in this comparison was the Latent Diffusion Model (LDM), which achieved a remarkable PSNR of 30.4 dB and an SSIM of 0.892. These results clearly demonstrate the LDM’s superior capability in preserving the structural and perceptual quality of the denoised images. Close behind the LDM were the SRGAN (Super-Resolution GAN) and Unet + GAN models, which delivered PSNR values of 29.9 dB and 30.0 dB, respectively, along with SSIM values of 0.881 and 0.884. These findings suggest that the synergistic combination of generative adversarial networks and convolutional neural networks can be highly effective in image denoising tasks. The WGAN-GP (Wasserstein GAN with Gradient Penalty) and DnCNN (Denoising Convolutional Neural Network) models also performed well, with PSNR values around 29.2 dB and SSIM values of 0.871. Meanwhile, the Denoising Autoencoder, PixelCNN, and Deep Image Prior models demonstrated slightly lower, yet still respectable, PSNR and SSIM values. At the lower end of the spectrum were the Convolutional Neural Network (CNN) and BM3D (Block-Matching and 3D Filtering) models, which exhibited the weakest performance with PSNR values of 27.7 dB and 28.3 dB, and SSIM values of 0.824 and 0.842, respectively. As illustrated in Fig. 7, the performance of missing data imputation techniques is visualized through a scatter plot, highlighting the relationship between Mean Squared Error (MSE) and R^2^. The Diffusion Model clearly stands out, positioned in the low MSE–high R^2^ region, indicating its superior accuracy. It achieves the lowest MSE (0.016) and the highest R^2^ (0.983), reinforcing its effectiveness in reconstructing missing data with minimal error. The Transformer-based model and BERT are also positioned favorably, exhibiting relatively low MSE values of 0.023 and 0.025, respectively, with R^2^ values exceeding 0.970.

**Table 8:** Comparison of missing data imputation techniques.

Model	MSE (W^2^/m^4^)	RMSE (W/m^2^)	MAE (W/m^2^)	*R* ^2^	MAPE (%)
BERT^67^	0.025	0.155	0.034	0.974	2.92
GPT-3^68^	0.029	0.168	0.036	0.970	3.08
Transformer-based^69^	0.023	0.149	0.032	0.977	2.58
HG-LSTM^70^	0.034	0.183	0.040	0.959	3.80
STA-GAN^71^	0.027	0.160	0.035	0.972	3.15
VAE^72^	0.030	0.171	0.037	0.966	3.50
BiLSTM^73^	0.032	0.177	0.039	0.961	3.68
Random forest^74^	0.039	0.196	0.043	0.948	4.10
KNN^75^	0.042	0.204	0.046	0.943	4.44
Matrix factorization^76^	0.036	0.188	0.041	0.953	3.92
Autoencode^77^	0.031	0.174	0.038	0.968	3.55
Diffusion model	0.016	0.122	0.023	0.983	1.47

Before the preprocessing step, as shown in Table 9, the “Our” model outperforms all other methods, including ANN, SCG, BR, SVR, MLP, traditional LSTM, and GRU. It achieves the lowest Mean Squared Error (MSE) of 10.721 W^2^/m^4^, Root Mean Squared Error (RMSE) of 3.084 W/m^2^, Mean Absolute Error (MAE) of 1.471 W/m^2^, and Mean Absolute Percentage Error (MAPE) of 10.853%. The R-squared (R^2^) value of 0.993 (unitless) further indicates that the “Our” model has the highest goodness of fit, demonstrating its superior ability to capture the complex underlying patterns in the solar radiation data.

The preprocessing step significantly enhances the performance of all models, as evident in Table 10. The “Our” model continues to excel, achieving the lowest MSE of 8.245 W^2^/m^4^, RMSE of 1.768 W/m^2^, MAE of 0.712 W/m^2^, and MAPE of 8.678%. Additionally, the R^2^ value improves to 0.996 (unitless), further validating the model’s remarkable predictive performance enhancement after preprocessing.

**Fig. 8:**
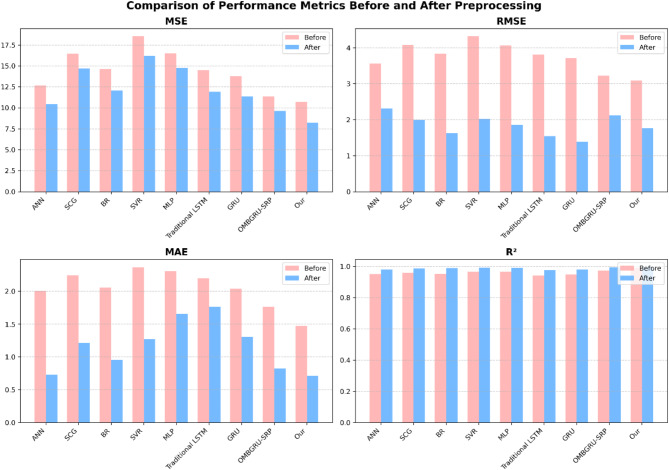
Comparison of solar radiation prediction before and after preprocessing layer.

Compared to the competing models, including ANN, SCG, BR, SVR, MLP, traditional LSTM, and GRU, the “Our” model demonstrates the most significant accuracy improvements post-preprocessing. This underscores its strong adaptability to solar radiation prediction tasks, highlighting its ability to leverage data preprocessing techniques effectively for superior prediction accuracy and robustness. Fig. 8 illustrates the comparative analysis of model performance before and after preprocessing.

**Table 9:** Results of solar radiation prediction before the preprocessing step.

Methods	MSE (W^2^/m^4^)	RMSE (W/m^2^)	MAE (W/m^2^)	*R* ^2^	MAPE (%)
ANN	12.682	3.562	2.008	0.951	14.674
SCG	16.478	4.071	2.245	0.960	16.134
BR	14.623	3.826	2.059	0.954	13.912
SVR	18.538	4.317	2.367	0.967	15.029
MLP	16.493	4.069	2.305	0.966	16.589
Traditional LSTM	14.508	3.812	2.197	0.943	18.354
GRU	13.798	3.712	2.038	0.950	14.923
OMBGRU-SRP	11.372	3.217	1.765	0.973	11.652
Our	10.721	3.084	1.471	0.993	10.853

**Table 10:** Results of solar radiation prediction after the preprocessing step.

Methods	MSE (W^2^/m^4^)	RMSE (W/m^2^)	MAE (W/m^2^)	*R* ^2^	MAPE (%)
ANN	10.426	2.315	0.728	0.982	12.395
SCG	14.712	1.995	1.211	0.987	13.472
BR	12.048	1.629	0.955	0.990	12.108
SVR	16.193	2.022	1.272	0.993	13.845
MLP	14.759	1.859	1.654	0.992	14.276
Traditional LSTM	11.926	1.543	1.764	0.978	16.782
GRU	11.367	1.389	1.302	0.981	12.964
OMBGRU-SRP	9.632	2.122	0.825	0.995	9.573
Our	8.245	1.768	0.712	0.996	8.678

## Conclusion and future work

This paper presented a hybrid model for prediction of solar radiation after combining the satellite images and the satellite data. The paper also presents a multi-layer preprocessing contains pixel and data imputation and removing noise step with novel modification of adding the 8-connected pixel for enhancing the integrity of pixel generation using GANs. The framework utilizes a hybrid approach, where the first path processes the satellite images by removing noise using a Latent Diffusion Model, imputing missing pixels with a modified RF + Identity GAN, and extracting informative features using a Self-Organizing Map. The second path focuses on the tabulated data, employing a Diffusion Model to impute missing values. The outputs from both paths are then merged, and feature selection is performed before feeding the data into an LSTM network for solar radiation prediction. The experiments conducted demonstrate the effectiveness of the proposed stages, such as missing pixel imputation, noise removal, and missing data imputation, in improving the overall accuracy of solar radiation prediction models. The results show that the hybrid approach, which combines the strengths of both satellite images and tabulated data, outperforms models relying on a single data source. While the current research presents a significant step forward in solar radiation prediction, there are several areas for future exploration and improvement. One potential direction is to explore advanced feature extraction techniques, such as investigating more sophisticated methods for extracting relevant features from satellite images, including deep learning-based feature extraction. This could further enhance the predictive capabilities of the framework by capturing more informative patterns from the satellite data. Additionally, incorporating additional data sources, such as meteorological data, ground-based measurements, and socioeconomic factors, could provide a more comprehensive understanding of the factors influencing solar radiation and improve the overall prediction accuracy. Evaluating the proposed framework in diverse geographical regions would also help assess its robustness and generalizability, ensuring its applicability across different climatic conditions. Furthermore, enhancing the framework to enable real-time solar radiation prediction and short-term forecasting could significantly benefit the planning and optimization of solar energy systems. Finally, exploring ways to improve the computational efficiency and scalability of the framework, particularly for large-scale satellite data processing, would enable its implementation in practical, real-world applications. By addressing these future research directions, the accuracy, reliability, and applicability of solar radiation prediction models can be further enhanced, ultimately contributing to the efficient utilization and management of this renewable energy resource.

## Data Availability

The datasets used and/or analyzed during the current study are available from the corresponding author upon reasonable request.

## Declarations

Correspondence and requests for materials should be addressed to Mohammed Attya.
